# Biological Functions and Potential Therapeutic Significance of O-GlcNAcylation in Hepatic Cellular Stress and Liver Diseases

**DOI:** 10.3390/cells13100805

**Published:** 2024-05-09

**Authors:** Zun Mao, Junpeng Mu, Zhixiang Gao, Shile Huang, Long Chen

**Affiliations:** 1 Jiangsu Key Laboratory for Molecular and Medical Biotechnology, College of Life Sciences, Nanjing Normal University, Nanjing 210023, China; zunmao@njnu.edu.cn (Z.M.); 221202075@njnu.edu.cn (Z.G.); 2 Department of Clinical Medicine, Xuzhou Medical University, Xuzhou 221004, China; y20240229@163.com; 3 Department of Biochemistry and Molecular Biology, Louisiana State University Health Sciences Center, 1501 Kings Highway, Shreveport, LA 71130-3932, USA; 4 Department of Hematology and Oncology, Louisiana State University Health Sciences Center, 1501 Kings Highway, Shreveport, LA 71130-3932, USA; 5 Feist-Weiller Cancer Center, Louisiana State University Health Sciences Center, Shreveport, LA 71130-3932, USA

**Keywords:** OGT, O-GlcNAcylation, hepatic cellular stress, inflammation, fibrosis, NAFLD, hepatocellular carcinoma

## Abstract

O-linked-β-D-N-acetylglucosamine (O-GlcNAc) glycosylation (O-GlcNAcylation), which is dynamically regulated by *O*-GlcNAc transferase (OGT) and *O*-GlcNAcase (OGA), is a post-translational modification involved in multiple cellular processes. O-GlcNAcylation of proteins can regulate their biological functions via crosstalk with other post-translational modifications, such as phosphorylation, ubiquitination, acetylation, and methylation. Liver diseases are a major cause of death worldwide; yet, key pathological features of the disease, such as inflammation, fibrosis, steatosis, and tumorigenesis, are not fully understood. The dysregulation of O-GlcNAcylation has been shown to be involved in some severe hepatic cellular stress, viral hepatitis, liver fibrosis, nonalcoholic fatty acid liver disease (NAFLD), malignant progression, and drug resistance of hepatocellular carcinoma (HCC) through multiple molecular signaling pathways. Here, we summarize the emerging link between O-GlcNAcylation and hepatic pathological processes and provide information about the development of therapeutic strategies for liver diseases.

## 1. Introduction 

Glucose metabolism is an indispensable part of hepatic cellular activity. When glucose is converted to fructose-6-phosphate (F6P), most of it enters the glycolytic pathway [1]. It is worth noting that about 2~5% F6P will enter the hexosamine biosynthetic pathway (HBP) to perform unique physiological functions [2]. The end product of HBP is uridine diphosphate N-acetylglucosamine (UDP-GlcNAc), which belongs to the important substrate of O-linked-β-D-N-acetylglucosamine glycosylation (O-GlcNAcylation) transferase (OGT) [3]. During the process of O-GlcNAcylation, OGT is capable of transferring a single GlcNAc, a vital moiety of the UDP-GlcNAc group, to serine (Ser) or threonine (Thr) residues on target proteins. UDP-GlcNAc is the nucleotide sugar donor for this modification [4]. On the contrary, O-GlcNAcase (OGA) is responsible for removing the GlcNAc moiety from O-GlcNAcylated proteins so as to inhibit the process of O-GlcNAcylation [5]. O-GlcNAcylation is a highly dynamic process regulated by OGT/OGA.

O-GlcNAcylation, particularly prevalent in the liver, is the core hub for controlling systemic glucose homeostasis due to its nutritional sensitivity and precise spatiotemporal regulation of insulin signal transduction [6]. The intracellular O-GlcNAcylation level is tightly controlled, and O-GlcNAcylation is a critical regulator of hepatic differentiation [7]. The pathology of various liver diseases has highlighted hepatic metabolic disorder and dysfunction, and abnormal O-GlcNAcylation also plays a specific pathological role in these processes [6]. OGT-mediated O-GlcNAcylation generally promotes cell survival to inhibit liver damage, but it may also promote cancer cell proliferation [8]. Correspondingly, the effect of downward adjustment is generally opposite to that of upward adjustment [8,9]. In addition, changes in glucose flux can also cause changes in the activity of the corresponding metabolic pathways such as HBP or OGT/OGA concentrations, resulting in altered levels of O-GlcNAcylation. High glucose (HG) will effectively enhance the activation of HBP, thus leading to increased O-GlcNAcylation levels of the related proteins [10]. At the same time, O-GlcNAcylation is responsible for influencing subsequent molecular pathways through its regulation of specific molecules, thus exerting final effects such as anti-apoptosis and anti-fibrosis [11,12]. Furthermore, interactions with specific cellular metabolism and other protein modifications are also important for O-GlcNAcylation the pathological changes in the liver. For instance, O-GlcNAcylation-related hepatocellular carcinoma (HCC) can be facilitated by HG [13,14]. Lipid metabolism, centered on fat synthesis, mainly affects the development of hepatic steatosis via an interaction with O-GlcNAcylation [15,16]. Other post-translational modifications, such as protein phosphorylation [17], acetylation [18], ubiquitination [19], and SUMOylation [20], can also play a synergistic or antagonistic role in the regulation of O-GlcNAcylation-mediated molecular pathways.

To facilitate the treatment of diseases, artificial intervention of HBP, OGT/OGA concentration, and other regulatory factors is needed. Although a large number of problems still need to be solved, great progress has been made in this area. Regarding this topic, this review will explore the relationship between O-GlcNAcylation and liver pathophysiology to predict future pharmaceutical development and propose new strategies for the treatment of liver diseases.

## 2. The Involvement of O-GlcNAcylation in Hepatic Cellular Stress

Cellular stress response refers to the cell’s defense against cell damage caused by environmental factors. When damaged, hepatocytes can repair themselves through certain processes like autophagy [21,22]. If the injured cells cannot be restored, apoptosis will remove them in a bid to protect the surrounding cells [22,23]. However, in high-intensity injury, cellular necrosis often occurs, which leads to tissue damage [23]. Although the liver has a great ability to repair itself, blindly suppressing cell death or self-repair could be harmful [24]. It has been shown that O-GlcNAcylation plays a vital role in inhibiting cell death. In addition, O-GlcNAcylation is also involved in the regulation of cell adaptation to various environmental changes, such as glucose deprivation [25]. Therefore, the understanding of related molecular mechanisms is of significance for the treatment of liver diseases.

**Figure 1:**
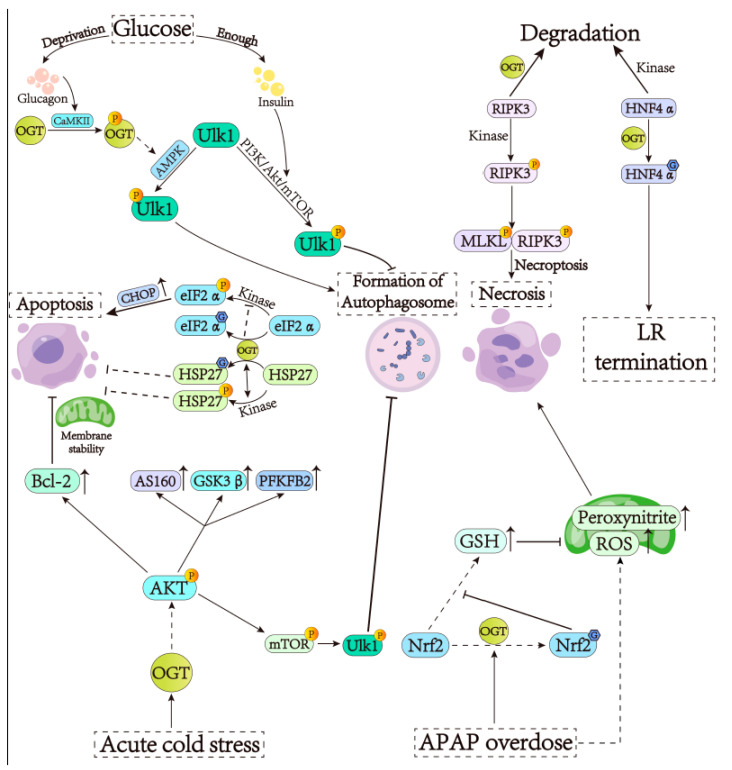
The involvement of OGT in hepatic cellular stress. (1) In the presence of adequate glucose, insulin activates the PI3K/AKT/mTOR pathway, leading to the phosphorylation of ULK1 on Ser757 to inhibit hepatic autophagy. In the deficiency of glucose, glucagon releases Ca^2+^ to activate CaMKII, leading to the phosphorylation of OGT, which can activate the AMPK-mediated phosphorylation of ULK1 on Ser317, Ser555, and Ser777 to promote hepatic autophagy. (2) O-GlcNAcylation can exert anti-apoptotic effects by inhibiting CHOP expression or promoting HSP27 expression. In ER stress, phosphorylated eIF2α is modified by O-GlcNAcylation to reduce CHOP expression and ultimately inhibit apoptosis. In contrast, O-GlcNAcylation modification can allow HSP27 to enter the nucleus, thus promoting HCC proliferation. (3) Phosphorylated RIPK3 recruits and phosphorylates MLKL, leading to necroptosis. O-GlcNAcylation promotes RIPK3 degradation and suppresses necroptosis. Similarly, O-GlcNAcylation of HNF4α also reduces self-phosphorylation to terminate LR. (4) OGT can activate AKT to help the survival of hepatocytes at a low temperature. AKT can increase Bcl-2 expression and maintain mitochondrial membrane integrity; it also can activate AS160, GSK3β, and PFKFB2 to promote glucose-related metabolism. (5) O-GlcNAcylation reduces the NRF2 activity induced by APAP, reduces GSH levels, and promotes liver injury.

Starvation can induce liver autophagy to provide nutrients for other vital organs like the brain and maintain whole-body homeostasis [26]. Hepatic autophagy is suppressed by insulin by virtue of the mammalian target of the rapamycin (mTOR) pathway [27,28]. The opposite is that under the hypoglycemia condition, glucagon promotes OGT phosphorylation by releasing Ca^2+^ to activate calcium/calmodulin-dependent kinase II (CaMKII); the phosphorylated OGT induces O-GlcNAcylation, which causes the phosphorylation of unc-51-like kinase 1 (ULK1) at different sites and subsequent liver autophagy [26]. Additional studies have shown that abundant nutrients can activate the PI3K/AKT/mTOR pathway to directly phosphorylate protein ULK1, thus suppressing autophagy [27,29] (Figure 1). In terms of cell death, O-GlcNAcylation has an overall inhibitory effect on the apoptosis and necrosis of hepatocytes; so, it plays an important role in anti-liver injury. With respect to apoptosis, researchers found that O-GlcNAcylation plays an anti-apoptotic role by inhibiting the function of C/EBP homologous protein (CHOP) or promoting the function of heat shock protein 27 (HSP27) [30]. CHOP is a transcription factor that regulates the expression of genes participating in apoptosis [31]. Under endoplasmic reticulum (ER) stress, the enhanced O-GlcNAc modification of eukaryotic translation initiation factor 2α (eIF2α) can downregulate the phosphorylation of eIF2α, thus reducing the expression of CHOP and ultimately inhibiting apoptosis [32]. As a molecular chaperone and anti-apoptotic protein [33,34], HSP27 phosphorylation status is closely linked to cancer progression. It is worth noting that O-GlcNAc modification can also facilitate HSP27 to enter the nucleus, thus promoting the proliferation of HCC cells [35]. Further studies have shown that OGT deficiency can worsen concanavalin A (Con A)-induced autoimmune hepatitis, which is associated with the impaired differentiation of regulatory T cells (Tregs) regulated by Notch signaling and induces cellular apoptosis [36]. As for cell necroptosis, O-GlcNAc modification, in particular, plays a cellular protective role against necroptosis. Necroptosis is a peculiar programmed cell death in the liver [37], which can lead to a range of acute and chronic liver disorders, such as alcoholic steatohepatitis and nonalcoholic steatohepatitis (NASH) [38,39]. During the process of necroptosis, the phosphorylated receptor-interacting protein kinase 3 (RIPK3) recruits and activates mixed lineage kinase domain-like (MLKL); the latter then translocates to the cytomembrane, leading to membrane rupture [40]. On the contrary, O-GlcNAc modification can reduce the stability of the RIPK3 protein and inhibit its expression, thus suppressing cellular necroptosis [8] (Figure 1).

Liver regeneration (LR) plays an important role in the adaptation to hepatic injury. LR is tightly regulated by redundant signals that control tissue remodeling as well as the initiation and termination of cell proliferation, and the defective termination of LR leads to reduced redifferentiation of hepatocytes, causing fibrosis, nodules, and even HCC [41]. Hepatocyte nuclear 4α (HNF4α), a nuclear receptor critical to hepatocyte differentiation and function, plays a crucial role in LR termination [42]. It was proposed that HNF4α is normally O-GlcNAcylated, which suppresses its degradation or cytoplasmic relocalization by inhibiting its phosphorylation [41] (Figure 1). Therefore, O-GlcNAc modification plays a vital role in terminating regeneration and preventing liver dysplasia.

In addition to protecting hepatocytes from damage and preventing excessive regeneration, O-GlcNAcylation also helps them adapt to different environmental changes. As discussed below, O-GlcNAc modification plays a comprehensive role in assisting hepatocytes to adapt to chemical and physical damage, revealing a new way for O-GlcNAcylation to protect against hepatic injury. As the synthesis pathway of UDP-GlcNAc, HBP belongs to the glucose-dependent metabolic pathway. Once hepatic glucose is deprived, the flux into HBP decreases accordingly, leading to downregulated O-GlcNAcylation levels, thus affecting the survival of liver cells [43]. It is worth noting that glucose deprivation can induce the upregulation of OGT expression and downregulation of OGA expression in HepG2 cells in the first few hours to compensate for reduced O-GlcNAc modification [25,44]. During this period, glycogen synthase (GS) is also O-GlcNAcylated, leading to reduced glycogen synthesis for antagonizing glucose deprivation [44]. Therefore, the regulation of OGT/OGA concentration in hepatocytes may be important for cellular adaptation to glucose deprivation and reduced HBP flux.

Cold, as a common environmental stress, causes increased heat production and accelerated metabolism and even affects its production performance. It has been demonstrated that the deletion of O-GlcNAcylation disrupts lipid metabolism, causes hepatic edema and fibrosis, and alters mitochondrial apoptosis. This modification can be made worse by cold induction [45]. In addition, O-GlcNAcylation is involved in the regulation of acute cold stress. It has been found that OGT can assist hepatocytes in surviving at low temperatures by activating protein kinase B (AKT) [46]. On the one hand, the expression of anti-apoptotic protein B-cell lymphoma 2 (Bcl-2) is increased by AKT to maintain mitochondrial membrane integrity [47]. On the other hand, the phosphorylation and activation of 160 kDa AKT substrate (AS160), glycogen synthase kinase-3β (GSK3β), as well as 6-phosphofructo-2-kinase/fructose-2,6-biphosphatase 2 (PFKFB2) promote glucose transport, glycogen synthesis, and glycolysis, respectively, so as to maintain intracellular energy balance to prevent apoptosis (Figure 1). In addition, AKT also inhibits autophagy through the mTOR-ULK1 pathway, which can be enhanced by O-GlcNAcylation [48,49]. Conclusively, O-GlcNAc modification suppresses autophagy and the apoptosis of hepatocytes to protect cells under cold stress.

Oxidative stress is also one of the important causes of liver cell death. Interestingly, O-GlcNAcylation appears to have a bidirectional regulatory effect on the resistance to the oxidative damage of hepatocytes. There is evidence that suggests that the long-term elevation of O-GlcNAcylation modulates mitochondrial function to reduce mitochondrial respiration and ROS production, thus downregulating the nuclear factor erythroid 2-related factor 2 (NRF2)-mediated oxidative stress response and protecting mitochondrial functions [50]. However, if the expression of OGA is not increased in the meantime, excessive reduction in mitochondrial respiration will have adverse effects on hepatic cells [50]. NRF2 is a regulator involved in glutathione (GSH) synthesis [51,52]. A study showed that O-GlcNAcylation reduces the activity and stability of NRF2 in acetaminophen (APAP)-induced liver injury, thereby reducing the antioxidant GSH level [53] (Figure 1). It should be noted that the increase in O-GlcNAcylation can promote the formation of peroxynitrite through protein adduction and mitochondrial oxidative stress. Peroxynitrite is a highly reactive oxidant involved in many diseases as strong oxidation can cause a free radical solution-mediated nitrification reaction, leading to damage to cells [54]. In addition, the O-GlcNAcylation-induced activation of the c-Jun N-terminal kinase (JNK) pathway was also found to promote hepatic injury by APAP elevation [53,55,56]. Hence, under certain circumstances, O-GlcNAcylation may also have adverse effects on cell survival.

## 3. O-GlcNAcylation Is Required for the Development of Infectious Liver Diseases

O-GlcNAcylation’s overall protective effect on hepatocytes is also reflected in assisting the fight against foreign infections. The corresponding pathogens are mainly the hepatitis B virus (HBV), the hepatitis C virus (HCV), and *Entamoeba histolytica* (*E. histolytica*), which will be discussed separately in the following content. Infectious liver injury caused by HBV and HCV is closely related to the subsequent development of liver cirrhosis, HCC, and other chronic hepatic diseases. Although the HBV infection in most adults is self-limiting, about 5–10% of infected adults and over 90% of newborns develop chronic infections [57]. Similarly, chronic HCV infection was also reported to be hard to cure in a subset of patients [58]. Of interest, studies have uncovered that O-GlcNAcylation plays an important role in fighting against HBV and HCV infections [59], which provides a new idea for the treatment of viral hepatitis.

**Figure 2:**
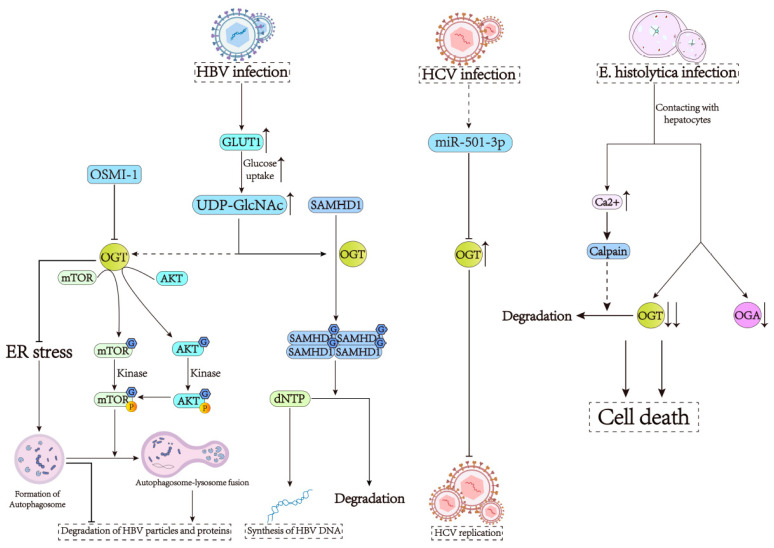
OGT is required for the development of infectious liver diseases. (1) HBV infection increases GLUT1 expression, promotes glucose uptake, provides a substrate for UDP-GlcNAc, and modifies SMDH1 by O-GlcNAcylation, thus inhibiting viral replication. In addition, cellular autophagy is activated after HBV infection. OGT affects autophagosome formation by affecting ER stress, AKT-mTOR signaling pathway, as well as autophagosome-lysosome fusion, and regulates HBV replication. (2) miR-501-3p can silence OGT to increase HCV infection. (3) *E. histolytica* makes contact with hepatocytes, which reduces OGT/OGA levels, increases Ca^2+^, activates calpain, cleaves OGT, and eventually leads to host cell death.

Sterile alpha motif and histidine/aspartic acid domain-containing protein 1 (SAMHD1) play important roles in host innate immunity against HBV, while its activation can be upregulated by HBV infection [60,61]. As a deoxy-ribonucleoside triphosphate (dNTPase), SAMHD1 restricts the synthesis of viral DNA by degrading dNTPs in cells, thereby inhibiting HBV replication [62]. The O-GlcNAcylation of SAMHD1 on Ser93 enhances its antiviral activity in the following ways [61]. First, HBV infection increases the expression of glucose transporter 1 (GLUT1) on the surface of hepatocytes, thereby promoting glucose uptake, which can meet the energy requirements of cells in response to a viral infection. However, this enhanced nutrient state also provides substrates for UDP-GlcNAc production, leading to increased protein O-GlcNAcylation [63,64]. Then, the OGT-mediated O-GlcNAcylation of SAMHD1 on Ser93 enhances the stability and tetramerization of SAMHD1, which is critical to its dNTPase activity to guard against the virus [61] (Figure 2). Aside from regulating the activity of SAMHD1, O-GlcNAcylation also influences cellular autophagy, a process impacting HBV replication and assembly. Autophagy is a powerful course that regulates the post-transcriptional steps of the HBV life cycle, especially enhancing HBV assembly and hepatitis B surface antigen (HBsAg) secretion. It has been described that HBV infection can activate autophagy for replication and envelopment [65,66]. However, after autophagosome–lysosome fusion, HBV particles and HBsAg undergo extensive autophagic degradation [67,68]. Promoting the formation of autophagosome and suppressing the fusion of autophagosome–lysosome could facilitate the secretion of HBsAg and suppress the degradation of HBsAg, which can increase the level of HBsAg and the levels of HBV relaxed circular DNAs (rcDNAs). A study has shown that the inhibition of OGT can significantly increase the formation of autophagosomes and HBV replication by triggering ER stress, AKT/mTOR inhibition, as well as blockade of autophagosome–lysosome fusion [59]. In summary, O-GlcNAcylation is indeed able to affect cellular autophagy, but further research on O-GlcNAcylation is required for a better understanding of the HBV–host interaction as well as the pathogenesis of HBV (Figure 2).

There is evidence that indicates that every step of the HCV replication cycle depends on specific virus–host interactions, which involve host proteins and miRNAs [69]. It has been shown that miR-501-3p can downregulate the expression of OGT at the protein level on the one hand, while the knockdown of OGT increases the infectivity and size of HCV particles on the other hand [70], suggesting a link between O-GlcNAcylation and miR-501-3p (Figure 2). However, given the fact that OGT expression increases in a patient’s liver tissue during the development and progression of liver diseases, OGT may play a dual role in HCV morphogenesis.

In addition to helping hepatocytes resist virus invasion, O-GlcNAcylation also plays an important role in anti-parasitic infections. *E. histolytica* is an intestinal protozoan and a tissue-invasive parasite that leads to amoebic colitis and, occasionally, liver abscesses in humans [71]. It possesses virulence factors, such as Gal/GalNAc lectin, amoebapore, and cysteine protease, and can induce host cell death in contact-dependent and -independent manners [72,73,74,75]. It has been reported that O-GlcNAcylation protects cells from cellular stress and cell death [76,77]. In contrast, decreased O-GlcNAc levels resulting from OGA overexpression could promote apoptotic cell death [78]. A recent study has found that *E. histolytica*-induced death signaling molecules in HepG2 cells are correlated with decreased cellular O-GlcNAc levels [71]. First, in HepG2 cells, the contact between *E. histolytica* and host cells is the key to O-GlcNAcylation, which leads to a dramatic reduction in O-GlcNAcylated protein in the cells, resulting in the death of host cells. The expression of OGT in host cells has been noticed to decrease after 5 min of co-incubation with *E. histolytica* [71], indicating that the OGT cleavage induced by the parasite plays a vital role in accelerating the death of host cells. Amoebas also come into contact with host cells via Gal/GalNAc lectin, leading to a rapid increase in intracellular Ca^2+^ and, in turn, the activation of calpain; pretreatment with calpeptin, a calpain inhibitor, could block OGT cleavage in host cells induced by *E. histolytica* [71,79,80] (Figure 2). These observations suggest that increasing OGT expression directly or indirectly may be a new approach to treating amebic liver abscesses.

## 4. O-GlcNAcylation Contributes to the Progression of NAFLD

Nonalcoholic fatty acid liver disease (NAFLD) is a kind of liver disease closely associated with liver nutrient metabolism. Both obesity and type 2 diabetes may lead to the accumulation of triglycerides in the liver, which can induce NAFLD [81]. Furthermore, nonalcoholic steatohepatitis (NASH) is considered to be the most severe form of NAFLD and a potential starting point for cirrhosis and HCC [82]. As a nutritional sensor, O-GlcNAcylation affects hepatic triglyceride accumulation by regulating upstream glucose uptake, downstream fatty acid synthesis, and others [83]. In addition, a faulty diet modulates the global O-GlcNAcylation of liver proteins, accompanied by decreased activation of adenosine 5′-monophosphate (AMP)-activated protein kinase (AMPK), which could exacerbate metabolic syndromes through fat accumulation in the liver [84]. Therefore, exploring the metabolic pathogenesis of NAFLD associated with O-GlcNAcylation is valuable for the treatment of this chronic hepatic disease. 

**Figure 3:**
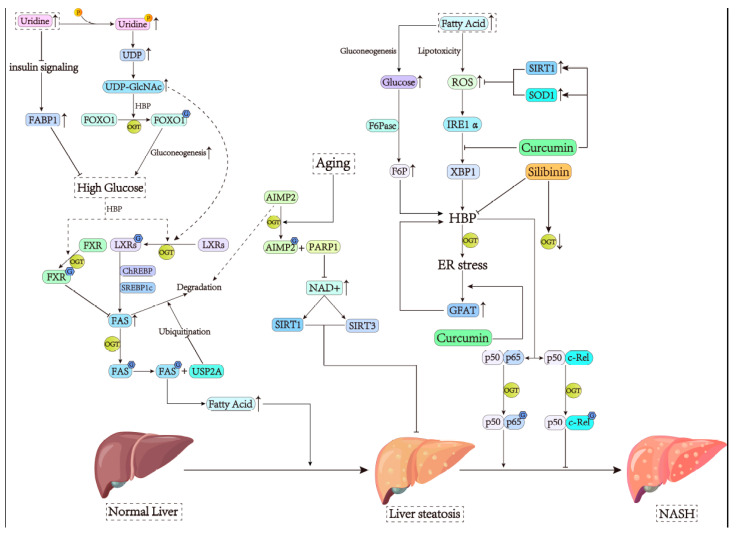
OGT contributes to the progression of NAFLD. (1) The phosphorylation of uridine increases UDP-GlcNAc levels, which is regulated by ChREBP and SREBP1C, modifying FAS by O-GlcNAcylation, and is later increased by an interaction with USP2A. Meanwhile, the increase in UDP-GlcNAc also modifies FOXO1 by O-GlcNAcylation, alters gluconeogenesis, i.e., the insulin pathway, and causes a decrease in FABP1, leading to the formation of NAFLD. In addition, the FAS inhibitor FXR, modified by O-GlcNAcylation, further inhibits the formation of NAFLD. (2) AIMP2 and PARP1 in aging fatty liver are associated with NAD^+^ levels. Higher levels of NAD^+^ increase the activity of SIRT1 and SIRT3, which can protect hepatocytes. (3) Excessive fat accumulation leads to the accumulation of ROS, triggers ER stress, and increases the expression of GFAT and OGT. XBP1 and FBPase are also involved in contributing to HBP-mediated inflammation. Of importance, curcumin and silibinin exert their anti-inflammatory effects by regulating the XBP1-related pathway as well as the expression of OGT.

It has been reported that after being modified by O-GlcNAc, fatty acid synthase (FAS) increases the interaction with the deubiquitinase ubiquitin-specific protease-2a (USP2A), thus reducing ubiquitination degradation and enhancing the expression of FAS for the increased synthesis of fatty acids [16]. Additionally, carbohydrate-responsive element-binding protein (ChREBP) and sterol regulatory element-binding protein 1c (SREBP1c), which regulate FAS expression, are both associated with O-GlcNAcylation’s indirect regulation of FAS. By modifying liver X receptors (LXRs), O-GlcNAcylation increases the transcription of ChREBP and SREBP1c, thereby upregulating FAS expression [85,86,87]. Meanwhile, a hyperglycemic environment was found to enhance the modification of ChREBP by O-GlcNAc to increase FAS expression [88]. The same effect was also uncovered in two lipogenic enzymes, namely, acetyl-CoA carboxylase (ACC) and stearoyl-CoA desaturase 1 (SCD1), and the glycolytic enzyme L-pyruvate kinase (L-PK), indicating an association between glucose metabolism and fatty acid synthesis [88] (Figure 3). Interestingly, FAS’s expression inhibitor farnesoid X receptor (FXR) can also be modified by O-GlcNAc to enhance its activity, suggesting that O-GlcNAc modification might have a dual effect on the regulation of fat in the liver [89,90]. 

Bisphenol A exposure has been gradually recognized as a risk factor for NAFLD [91]. Recent studies have shown that the NOD-like receptor family pyrin domain-containing 3 (NLRP3) inflammasome, as the core part of the inflammatory response, mediates the occurrence and development of NAFLD [92]. It has been shown that bisphenol A could induce NAFLD by promoting the O-GlcNAcylation of NLRP3 [93]. In addition, uridine can be utilized in combination with other drugs, such as zalcitabine and fenofibrate [94,95], to inhibit hepatic steatosis. However, the long-term usage of uridine alone can aggravate hepatic steatosis [96]. The phosphorylation of uridine is known to be capable of generating uridine diphosphate (UDP), one of the raw materials for UDP-GlcNAc synthesis. Studies have demonstrated that chronic uridine feeding not only promotes O-GlcNAc modification of forkhead box protein O1 (FOXO1) to increase gluconeogenic gene expression but also reduces the insulin pathway activity to decrease the expression of a liver-specific fatty acid binding protein 1 (FABP1), resulting in impaired glucose clearance and increased fasting blood glucose level [64,96,97]. Considering that the HG environment is one of the factors promoting NAFLD [87,88], the above observations suggest that caution is needed during the uridine treatment of hepatic steatosis (Figure 3).

Aging-related NAFLD is another type of liver disease that should not be ignored. Nowadays, the high prevalence of NAFLD in the elderly has become one of the factors affecting the average life expectancy of humans [98]. Reduced nutrient-sensing activity and metabolic disorder are the main features of aging and NAFLD [99,100]. A recent study has revealed that aging-related fatty liver induced by the dysregulation of O-GlcNAcylation is closely associated with aminoacyl tRNA synthetase complex-interacting multifunctional protein 2 (AIMP2) and poly (ADP-ribose, PAR) polymerase 1 (PARP1) [101]. AIMP2 can induce an increase in PARP1 hyperactivation in the liver of aged mice, possibly via a direct protein–protein association, leading to PAR accumulation. O-GlcNAc signaling has also been found to be significantly activated in aging-related fatty liver to reduce AIMP2 degradation [101]. Considering that PARP1 is the most abundant of the PARP family of enzymes in response to DNA damage during aging [102], it has been speculated that an overactivated AIMP2–PARP1 axis may partially contribute to NAD^+^ depletion [101]. Higher NAD^+^ levels can enhance the activity of sirtuin 1 (SIRT1) and sirtuin 3 (SIRT3), which have been reported to protect mice from fatty liver diseases such as NAFLD [103,104]. This also suggests that hepatic steatosis is more likely to occur in the aged liver due to reduced NAD^+^ as well as the hyperactivation of AIMP2–PARP1 (Figure 3). Therefore, O-GlcNAcylation plays an important role in contributing to aging-related hepatic steatosis.

An increased expression of sodium-glucose cotransporter 2 (SGLT2) and O-GlcNAcylation in hepatocytes can drive NASH [105]. In addition, deregulated hyper-O-GlcNAcylation favors NAFLD progression by reducing mitochondrial oxidation and promoting hepatic lipid accumulation. Hepatocyte-specific OGT downregulation can ameliorate NASH by improving mitochondrial functions [106]. Due to lipotoxicity, fat deposited in hepatocytes may induce inflammation via various pathways, causing the progression of NAFLD to NASH and increasing the risk of patients developing cirrhosis and even HCC [107]. Intriguingly, O-GlcNAcylation seems to promote not only the onset of NAFLD but also its progression to NASH. A previous study has shown that the NF-κB pathway is the primary inflammatory pathway in animal models of obesity [108]. The NF-κB dimer is composed of p50 and p65 subunits, and if the latter is modified by O-GlcNAc, it will release from the separation of IκB, resulting in increased nuclear translocation of NF-κB, followed by hepatic inflammatory damage [109]. Studies have found that lipotoxicity causes ROS accumulation and subsequent ER stress [110]. It has been shown that ER stress can upregulate the transcription of glutamine F6P amidotransferase (GFAT) and OGT, thereby increasing O-GlcNAc modification, eventually leading to the development of inflammation [111]. In this process, the transcription factor X-box-binding protein 1 (XBP1), as the downstream transcription factor inositol requiring enzyme 1α (IRE1α), is involved in the inflammatory response mediated by ER stress [112,113] (Figure 3). This effect has been confirmed in mice with a methionine–choline-deficient (MCD) diet [15]. Additionally, by increasing the F6P level and HBP flux, fructose-1,6-bisphosphatase (FBPase) is also involved in NASH induced by activated NF-κB, as detected in mice with an MCD diet [114]. This might be one of the crossovers between NASH and HG [115].

Of note, two potential drugs for NASH treatment have been studied. Curcumin, a natural phytopolyphenol pigment from *Curcuma longa*, has been found to block HBP through the downregulation of XBP-associated IRE1α and GFAT expression on the one hand and upregulate the expression of SIRT1 and superoxide dismutase 1 (SOD1) through O-GlcNAcylation signaling on the other hand, thus exerting its anti-inflammatory effect [15]. The second drug, silibinin, which is a major active constituent in the extract of *Silybum marianum,* blocks the nuclear translocation of NF-κB p65 by inhibiting OGT so as to exert its anti-inflammatory effect [114]. It would be interesting to determine whether the combination of the two drugs is more effective at treating NASH. 

## 5. O-GlcNAcylation Has a Dual Influence on Liver Fibrosis

Chronic liver injury caused by various factors can result in hepatic fibrosis and even liver cirrhosis. Due to severe complications such as gastrointestinal bleeding and HCC, liver cirrhosis has become a leading cause of mortality worldwide [116]. In the pathogenesis of liver fibrosis, the activation of hepatic stellate cells (HSCs) is the key, which is also pivotal in the antifibrotic or fibrotic effect of O-GlcNAcylation [117]. 

**Figure 4:**
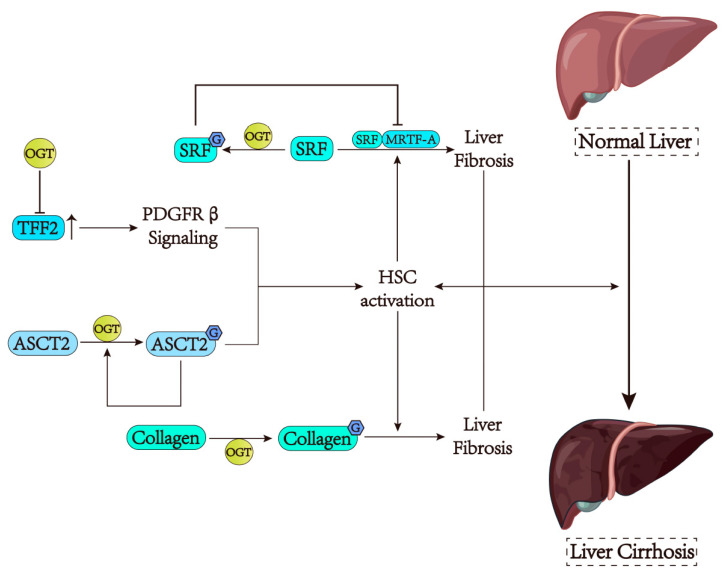
OGT has dual effects on liver fibrosis. (1) Hepatic stellate cell (HSC) is the key to activating liver fibrosis. After liver injury, SRF interacts with MRTFA to promote liver fibrosis. SRF suppresses this process when it is modified by O-GlcNAcylation. (2) OGT-deficient hepatocytes can secrete TFF2, which promotes HSC activation via the PDGFRβ pathway. Similarly, collagen modified by O-GlcNAcylation also promotes HSC activation and enhances fibrosis. (3) The O-GlcNAcylation modification of ASCT2 promotes the decomposition of Gln, thus promoting the development of liver fibrosis.

When liver injury occurs, HSCs can be activated into myofibroblast cells. During this period, the serum response factor (SRF), a pleiotropic transcription factor, increases the expression of the fibrogenic gene in HSCs (especially activated HSCs) by interacting with the cofactor myocardin-related transcription factor A (MRTF-A), thus promoting the development of hepatic fibrosis [118,119]. In mice with hepatic fibrosis, lower O-GlcNAcylation levels were detected in activated HSCs [12]. O-GlcNAcylated SRF was found to suppress the SRF-mediated transcription of the fibrogenic gene, especially α smooth muscle actin (α-SMA), thus preventing the activation of HSCs [12] (Figure 4). Similarly, another study, which started with dead mouse hepatocytes, discovered that O-GlcNAcylated proteins leaked from damaged hepatocytes can also induce antifibrotic activity by inhibiting the expression of α-SMA and other molecules [120]. Interestingly, OGT-deficient necroptotic hepatocytes can secrete trefoil factor 2 (TFF2), which promotes HSC activation, proliferation, and migration through platelet-derived growth factor receptor β (PDGFR β) signaling [121]. This also greatly increases the possibility of hepatic fibrosis (Figure 4). Of note, O-GlcNAcylation can also promote hepatic fibrosis. Collagen, which is also secreted from activated HSCs, can enhance its fibrotic effect by the modification of GlcNAc [122]. In addition, glutaminolysis is another important step in HSC activation [123]. A study reported that a higher level of alanine/serine/cysteine transporter 2 (ASCT2) modified by O-GlcNAc at Thr122 can promote its stability, membrane localization, and interaction with OGT, thus coordinating glutaminolysis in activated HSCs [124] (Figure 4). Taken together, OGT-mediated O-GlcNAcylation may have dual influences on liver fibrosis.

## 6. The Biological Function of O-GlcNAcylation in HCC

Hepatocellular carcinoma (HCC) is a major malignant tumor of the liver and a leading cause of mortality in humans [125]. HCC is caused by a variety of risk factors, including HBV or HCV infection, liver steatosis, diabetes, and other oncogenic factors [126]. Growing evidence suggests that the progression of HCC can be triggered by changes in gene expression in certain signaling pathways and is associated with alterations in cell cycle regulation [127]. Therefore, identifying new molecular mechanisms in the development of liver cancer may provide novel strategies for the diagnosis and treatment of the disease. OGT is highly expressed in HCC and associated with HCC development [128]. The levels of O-GlcNAcylation are also elevated in patients diagnosed with recurrent liver cancer [129], demonstrating that OGT is probably a new therapeutic target in HCC. In view of the complex effects of O-GlcNAcylation on HCC progression, this section is divided into carcinogenesis, proliferation, metastasis, recurrence of HCC, and two other parts.

### 6.1. The Role of O-GlcNAcylation in the Carcinogenesis of HCC

**Figure 5:**
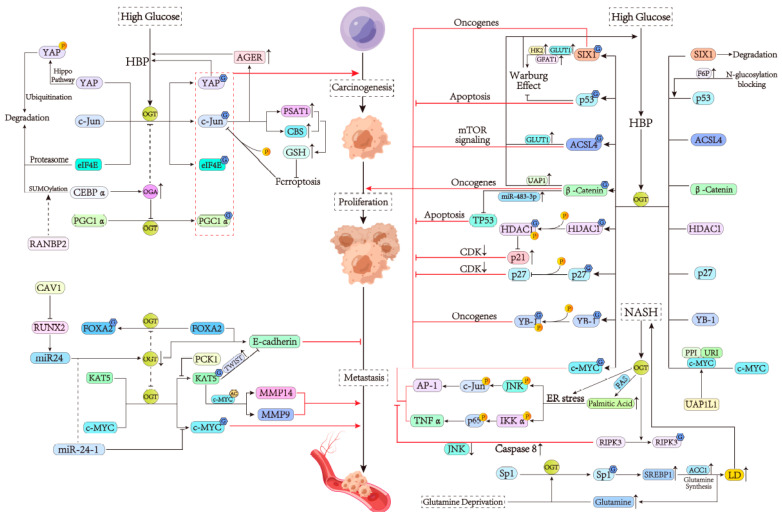
The biological function of OGT in HCC. (1) AGER enhances the O-GlcNAcylation modification of the proto-oncoprotein c-Jun, promoting HCC occurrence. However, the activation of ferroptosis forms a negative feedback pathway to inhibit the O-GlcNAcylation of c-Jun. The O-GlcNAcylation modification of YAP, eIF4E, and PGC1α promotes HCC progression. (2) The O-GlcNAcylation of ACSL4 activates the mTOR pathway to promote HCC proliferation. With the involvement of UAP1, O-GlcNAcylation modification occurs in β-catenin to promote HCC proliferation but increases the expression of the oncogene miR-483-3p and inhibits TP53-dependent cell apoptosis. SIX1 and F6P under the Warburg effect regulate the HBP pathway and increase the expression of related oncogenes, promoting HCC proliferation. After O-GlcNAcylation modification, the CDK inhibitors p21 and p27 lose the inhibitory activity on CDKs, promoting HCC proliferation. HG affects OGT activity and O-GlcNAcylation. OGT promotes ER stress induced by FARS and activates the JNK-related signaling pathway to promote HCC proliferation. RIPK3 inhibits the cleavage of caspase-8, JNK activation, and HCC proliferation, while RIPK3 is modified by O-GlcNAcylation. O-GlcNAc modification affects the expression of Sp1, SREBP1, and other genes to promote HCC proliferation, which is related to the accumulation of LD. (3) The downregulation of E-cadherin is a hallmark of cancer metastasis. O-GlcNAcylation of FOXA2 can reduce the transcription of E-cadherin and promote HCC metastasis after the same result of KAT5 and O-GlcNAcylation modification. miR-24-1 restricts OGT, but CAV1 can reduce the expression of RUNX2, lead to decreased miR24 transcription, relieve OGT restriction, and promote HCC metastasis.

Glucose metabolism is closely related to the activation of oncogenes and the malignant phenotype of HCC cells. It has been shown that a hyperglycemic environment in the liver increases HBP flux [130]. Upregulated O-GlcNAcylation then activates specific oncogenes, which can in turn promote carcinogenic effects. Among various stimulators, advanced glycosylation end product-specific receptor (AGER) was found to specifically increase the O-GlcNAcylation of the proto-oncoprotein c-Jun, thereby enhancing its activity and stability to accelerate tumorigenesis in HCC cells [14]. Interestingly, the activation of this pathway is persistent because the O-GlcNAcylation of c-Jun is capable of increasing AGER expression, thus forming a positive feedback loop, suggesting that targeting AGER may be a promising approach for HCC [14]. As for HG promoting HCC development, another positive feedback loop was also found. Yes-associated protein (YAP), as a potent oncogenic factor, is normally phosphorylated by the Hippo pathway at Ser127 to lead to its cytoplasmic localization and subsequent ubiquitination degradation [131,132]. Interestingly, an increased level of O-GlcNAcylated YAP at Thr241 in the HG environment can enhance YAP carcinogenic activity by antagonizing Ser127 phosphorylation [10]. Furthermore, stabilized YAP also forms a positive feedback loop by upregulating glucose uptake, the synthesis of metabolites used in HBP, and protein O-GlcNAcylation [10]. This suggests that targeting YAP may also be an effective strategy for HCC treatment. In addition, HG can also promote a specific form of carcinomatosis in HCC cells, known as stem-like cell potential. Mechanically, eukaryotic initiation factor 4E (eIF4E), a translation factor, binds to the stem-related gene *Sox2* 5′-untranslated region, and this process can be blocked by the degradation of eIF4E through a proteasome pathway [133]. Nevertheless, under the HG environment, the O-GlcNAcylation of eIF4E at Thr168 and Thr177 was found to protect it from degradation, thus promoting the malignant phenotype of HCC cells [134]. In summary, the fact that glucose upregulates HBP flux to activate oncogenes partially confirms that hyperglycemia is a risk factor for HCC (Figure 5).

Apart from HG, other factors can also regulate HCC carcinogenesis. Unlike the positive regulation of HG, ferroptosis involves the negative regulation of the proto-oncoprotein c-Jun [135], hence regulating HCC carcinogenesis. Stimulated ferroptosis promotes the phosphorylation of c-Jun and inhibits the modification of c-Jun by O-GlcNAc, while the overexpression of c-Jun modified by O-GlcNAc can stimulate GSH synthesis by increasing the transcription of phosphoserine aminotransferase 1 (PSAT1) and cystathionine-beta-synthase (CBS), thereby suppressing ferroptosis, deregulating the negative regulatory state of c-Jun, thereby promoting HCC development [135]. Accordingly, it can be concluded that the proto-oncoprotein c-Jun, as a key regulator, is effectively activated under HG conditions and suppressed under ferroptosis conditions, suggesting that c-Jun is a valuable target for HCC treatment. Similar to c-Jun, the oncogenic protein peroxisome proliferative-activated receptor gamma coactivator 1 alpha (PGC1α) also promotes the malignant phenotype of HCC through O-GlcNAcylation [136]. CCAAT/enhancer-binding protein alpha (CEBPα), as a transcriptional factor, can enhance OGA transcription without affecting OGT expression to suppress the hyper-O-GlcNAcylation of protein substrates such as PGC1α [20]. However, the RAN-binding protein 2 (RANBP2) promotes the SUMOylation and degradation of CEBPα by directly interacting with this molecule, thus augmenting the carcinogenesis of HCC [20]. SUMOylation is a multi-step reaction, which is similar to the cascade reaction of ubiquitination [137]. Overall, for the research on the molecular mechanism of HCC tumorigenesis, upstream regulatory factors and downstream carcinogenic factors are both required to be considered (Figure 5).

### 6.2. The Effect of O-GlcNAcylation on the Proliferation of HCC

In contrast to carcinogenesis, which depends on proto-oncogenes, HCC cell proliferation is more dependent on the factors that promote cell division and survival. A study has shown that GLUT1 acts as a membrane glucose transporter to increase HBP flux [138]. Acyl-CoA ligase 4 (ACSL4), which was reported to be overexpressed in HCC [139], enhances GLUT1-mediated O-GlcNAcylation [140]. However, upregulated O-GlcNAcylation can in turn increase the expression of ACSL4, and the latter inhibits HCC apoptosis via the activation of the mTOR signaling, thus forming a positive feedback loop for HCC cell proliferation [140]. In addition to controlling glucose uptake, key enzymes of the HBP are also potential targets for treating HCC, especially GFAT and uridine 5′-diphosphate (UDP)-N-acetylglucosamine pyrophosphorylase 1 (UAP1). The pharmacological inhibition or knockout of GFAT was found to increase the sensitivity of HCC cells to diamide-induced oxidative stress, indicating that combining targeting at GFAT with the induction of oxidative stress could achieve better therapeutic effects [9]. UAP1 is directly involved in the synthesis of UDP-GlcNAc [141]. On the one hand, UAP1 can increase the expression and stability of β-catenin through O-GlcNAcylation; on the other hand, UAP1 creates a new positive feedback loop [13]. Given that the nuclear accumulation of β-catenin leads to the continuous proliferation of HCC cells through the inhibition of apoptosis [142], UAP1 may be another potential target for HCC treatment (Figure 5). 

HCC cells can also promote HBP flux via the Warburg effect, which is one of the important means for cancer cells to ensure their survival [143]. Under this effect, tumor cells consume large amounts of glucose through anaerobic respiration to boost lactic acid production without generating enough energy so as to create a microenvironment only suitable for cancer cells to survive. High uptake of glucose by the Warburg effect also correspondingly increases HBP flux and subsequent protein O-GlcNAcylation [144]. As an important promoter of the Warburg effect, the transcription factor sine oculis homeobox homolog 1 (SIX1) can activate the expression of certain glycolytic and HBP genes such as *GLUT1* and *GFAT1* by interacting with the histone acetyltransferase HBO1 and AIB1 [145]. Upregulated O-GlcNAcylation at Thr276 inhibits the ubiquitination of SIX1, thereby enhancing its transcriptional activity [19]. Considering that SIX1 can strengthen the expression of oncogenes such as *c-MYC* to enhance HCC cell proliferation [146,147], this newly discovered positive feedback loop reveals a novel mechanism to promote HCC cell survival (Figure 5). Excessive glucose uptake by the Warburg effect is not only used for glycolysis and HBP but is also shunted by N-glycosylation pathways. The intermediate substrate of glycolysis F6P is a pivotal molecule in these three pathways. It has been found that once the flow of F6P to the N-glycosylation pathway is cut off, the excessive accumulation of F6P will further increase HBP flux, and then, the tumor suppressor p53 will be modified by O-GlcNAc to promote its stability [148]. Stable p53 can not only promote the apoptosis of HCC cells [149] but can also inhibit the Warburg effect by suppressing glycolysis [150]. In fact, normally, the N-glycosylation pathway of tumor cells is probably not blocked; so, the O-GlcNAcylation level of p53 may be lower than that of SIX1. Further research is needed to confirm or disprove this conjecture. If the accumulation of Fru6P does promote apoptosis of HCC cells, it will provide new insights into the treatment of HCC (Figure 5).

Previously, we mentioned that the accumulation of β-catenin can inhibit apoptosis, leading to HCC cell proliferation. Here, we discuss how β-catenin under HG conditions suppresses TP53-dependent apoptosis. After O-GlcNAc modification promoted by HG, β-catenin increases the expression of oncogene *miR-483-3p*, which targets the pro-apoptotic gene *BBC3 or PUMA* to suppress TP53-dependent apoptosis [151]. In addition, the tumor suppressor p21 is also involved in HG-mediated HCC cell proliferation. As a cyclin-dependent kinase (CDK) inhibitor, p21 is responsible for negatively regulating cell cycle progression [152]. Its transcription is regulated by histone deacetylase-1 (HDAC1) [153]. A recent study has found that in HepG2 cells, O-GlcNAcylation at Thr114 and Ser263 of HDAC1 enhances its phosphorylation and subsequent activation so as to downregulate p21 transcription via deacetylating histones [154] (Figure 5). The HG environment can promote this process, while a low-glucose environment has the opposite effect, suggesting that at least HG plays an important role in the inhibition of p21 [154]. It is worth noting that HDAC1-induced HCC progression relies on the mutual promotion of O-GlcNAcylation and phosphorylation. Another CDK inhibitor, p27, can be directly modified by O-GlcNAc to promote its phosphorylation [155]. Phosphorylated p27 will enter the cytoplasm to be degraded, thus facilitating the progress of the HCC cell cycle [156].

Although HG upregulates O-GlcNAcylation mainly by promoting UDP-GlcNAc synthesis, it can also do so by affecting OGT activity. A recent study has shown that the unconventional prefoldin RPB5 interactor (URI) acts as a rheostat to regulate OGT activity in response to different glucose concentrations [157]. Sufficient glucose induces the formation of the heterotrimeric URI/OGT/PP1γ complex to maintain OGT activity, thus stabilizing c-MYC via O-GlcNAc modification. On the contrary, glucose deprivation promotes the activation of cyclic AMP (cAMP)-dependent protein kinase A (PKA) and phosphorylation of URI, resulting in the release of PP1γ and subsequent inhibition of OGT by URI to degrade c-MYC (Figure 5). As an oncogene, c-MYC is able to induce human tumorigenesis. When activated in hepatocytes, c-MYC accelerates the proliferation of HCC cells. This uncovers a c-MYC-dependent adaptive survival response of tumor cells to glucose fluctuations. Similar to glucose, UAP1-like-1 (UAP1L1) can also upregulate O-GlcNAcylated c-MYC level by activating OGT, thus promoting the proliferation of HCC cells [158]. Intriguingly, UAP1L1 shows about 59% sequence identity to UAP1 but does not seem to participate in HBP [158]. The specific relationship between UAP1L1 and HBP still needs to be further explored.

Apparently, HG is very important for HCC cell proliferation, but NASH cannot be ignored either, which is featured by the activation of inflammatory signaling pathways. It has been demonstrated that OGT promotes ER stress by upregulating FAS-mediated palmitic acid synthesis, thus activating oncogenic JNK/c-Jun/AP-1 and NF-κB cascades [159]. In addition, the mutual suppression of RIPK3 and caspase 8 is also essential for the progression of NASH and HCC [39,160]. RIPK3 prevents HCC cell proliferation by inhibiting caspase 8 cleavage and subsequent JNK activation [160]. O-GlcNAcylated RIPK3 activity and expression decrease, promoting HCC development [8] (Figure 5). RIPK3 is a necroptosis promoter [8]; so, inhibiting RIPK3 may also facilitate HCC development by suppressing the necroptosis of tumor cells. Additionally, changes in glutamine (Gln) may also allow for the alteration of fat in HCC cells. Specifically, after Gln deprivation, O-GlcNAcylated specificity protein 1 (Sp1) is more capable of upregulating SREBP1 expression in the nucleus, and SREBP1 in turn promotes ACC1 expression and Gln synthesis, respectively, thus increasing the synthesis of Gln and later lipid droplet (LD) [161] (Figure 5). Notably, Gln can promote the O-GlcNAcylated Sp1 level, and the resulting positive feedback loop may accelerate the process of fat accumulation, thus aggravating NASH-related HCC [161]. Therefore, Gln also appears to be an effective target for the treatment of NASH.

### 6.3. The Regulation of O-GlcNAcylation in the Metastasis of HCC

As the most important feature of metastatic HCC cells, epithelial-mesenchymal transition (EMT) is accompanied by the downregulation of epithelial phenotype genes and upregulation of mesenchymal phenotype genes [162]. It is worth noting that the downregulation of E-cadherin is regarded as one of the markers of cancer cell migration. Therefore, E-cadherin plays an inhibitory role in HCC cell metastasis [163]. In contrast, O-GlcNAcylation decreases the transcription expression of E-cadherin by destabilizing transcription factor forkhead box protein A2 (FOXA2) [164]. There is also evidence that O-GlcNAcylation can facilitate HCC cell migration with E-cadherin downregulation in HG and NASH environments [154,159] (Figure 5). In addition, changes in other signature molecules of HCC metastasis, like the matrix metalloproteinase (MMP) family and vimentin [129,159], are often detected together with E-cadherin.

Some upstream molecules can specifically regulate HCC cell migration by affecting O-GlcNAcylation. Caveolin-1 (CAV1), a major structural protein of caveolae, can lead to the transcriptional attenuation of microRNA24 (miR24) by reducing runt-related transcription factor 2 (RUNX2) expression, thereby releasing miR24′s inhibition of OGT expression and promoting HCC cell migration through changes in E-cadherin and other substances [165]. Another study has demonstrated that O-GlcNAcylated lysine acetyltransferase 5 (KAT5) augments TWIST1 expression through the acetylation of histone H4, contributing to a decrease in E-cadherin [18]. The same study has also shown that O-GlcNAcylated KAT5 can promote the expression of HCC metastasis-promoting factors MMP9 and MMP14 by acetylating the oncoprotein c-MYC (Figure 5). In addition, phosphoenolpyruvate carboxykinase 1 (PCK1), a key enzyme of hepatic gluconeogenesis, inhibits HCC cell migration by suppressing the O-GlcNAc modification of KAT5 and the two subsequent pathways [18]. The upstream and downstream relationships between KAT5 and E-cadherin are important for the future treatment of HCC. Interestingly, miR24 and c-MYC have a direct link between each other. As an OGT suppressor, the miR24 subtype miR-24-1 was found to block HCC migration by reducing the O-GlcNAcylation and stability of c-MYC [166] (Figure 5). This discovery is a new addition to the RUNX2/miR24/OGT pathway mentioned above.

### 6.4. The Influence of O-GlcNAcylation on the Progression and Recurrence of HCC

The O-GlcNAcylation signaling pathway is simultaneously involved in multiple aspects of HCC progression. In general, the multifaceted promotion of HCC is mainly due to the diversity of activated oncogenes. For instance, in ribosomes, the O-GlcNAcylation of Ser122 promotes the stability of the receptor for activated C-kinase 1 (RACK1), which in turn enhances its interaction with protein kinase C βII (PKC βII) to result in eIF4E phosphorylation and activation, ultimately facilitating the translation of multiple oncogenes as well as cell proliferation, survival, migration, and invasion in HCC cells [167]. A similar effect was found in another study of the miR-15a/OGT/EZH2 axis. The overexpression of p53 was found to increase the expression of miR-15, an inhibitor of OGT expression, to reduce the stability of the enhancer of zeste homolog 2 (EZH2) and the subsequent expression of various oncogenes, thus hindering the progression of HCC in proliferation, migration, and invasion [11]. Like miR-15a, miR-424-5p is also a microRNA involved in the inhibition of HCC and was found to bind to the 3′-UTR of OGT to inhibit its expression, thereby reducing the stability of RAF1 and RAF1-mediated HCC progression [128]. Overall, the progression of HCC results from a combination of multiple targeted molecules and is a highly dynamic process; drugs targeting these molecules may simultaneously inhibit HCC progression from multiple aspects, which is of great value in treating HCC.

O-GlcNAcylation-mediated HCC recurrence is particularly associated with thermal ablation and liver transplantation (LT). Thermal ablation is a current form of tumor therapy that can apply heat energy to tissues, resulting in the necrosis of tumor cells. Inadequate thermal ablation was found to induce O-GlcNAc-modified hypoxia-inducible factor 1α (HIF-1α), which triggers a stronger Warburg effect, thereby achieving thermal tolerance of the remaining HCC cells and leading to tumor recurrence [168]. It should be noted that because HCC may continue to develop into new cancer, the treatment of HCC with thermal ablation is only temporary, and the really effective method is still LT. Therefore, thermal ablation usually acts as a “bridge” in LT. In terms of LT, a low expression of OGA is thought to promote tumor recurrence of HCC after LT, especially in patients with low levels of alpha-fetoprotein (AFP) [129]. In conclusion, O-GlcNAcylation-mediated HCC recurrence is closely related to the pathogenesis of HCC cell tolerance after therapy.

In view of the overall promoting effect of O-GlcNAcylation on HCC, the utilization of the OGT inhibitor OSMI-1 is, to some extent, of medicinal value. A previous study found that OSMI-1 is synergistic with doxorubicin (DOX), promoting the apoptosis of HepG2 cells. Interestingly, DOX increases the p53 level by inducing oxidative stress [169] but can simultaneously activate the NF-κB-related resistance pathways. The addition of OSMI-1 overcomes this drawback by suppressing the activation of NF-κB mediated by OGT [170]. In addition, the inhibition of OGT can also activate the apoptotic pathway via ER stress, thus synergizing the apoptotic effect of DOX [170].

In addition, other agents targeting OGT have been investigated. Corosolic acid can promote OGT phosphorylation and restrain YAP expression by inhibiting CDK19, thus blocking HG/HBP/YAP-mediated HCC cell proliferation [171]. Curcumin, as mentioned above, has been studied for treating NAFLD and NASH as well as NASH-associated HCC [15]. Since O-GlcNAcylation-mediated oncogenesis and proliferation of HCC cells are closely related to HG, the inhibitors of GLUT1 and GFAT could also have potential anticancer effects [9,140,172]. However, the development of new therapies still requires more thorough studies of the relevant signaling pathways to avoid potential drug resistance.

## 7. Conclusions

OGT-mediated O-GlcNAcylation contributes to the development of liver diseases by regulating various molecular signaling pathways. In recent years, growing evidence from in vitro and in vivo studies suggests that O-GlcNAc modification is indispensable for liver metabolism and pathological changes. As the source of UDP-GlcNAc, the HBP pathway is one of the important branches of hepatic glucose metabolism and is involved in the mediation of various pathways in the liver; the dysregulation of this pathway is associated with almost all liver diseases, such as liver injury, liver infections, and liver tumors, which confirms the importance of O-GlcNAc modification for the basic metabolism of the liver. In addition, the activities of key enzymes, namely, OGT and OGA, are also regulated by various factors, which constitutes another factor affecting O-GlcNAcylation levels. Thus, O-GlcNAcylation modification is a complex dynamic regulatory process in which the interactions of multiple signaling molecules or pathways cause a conformational change in downstream target molecules, finally altering the activities of related cells. 

O-GlcNAcylation in the liver involves many signaling molecules and pathways. Exogenous molecules, such as curcumin [15], acetaminophen [53], and corosolic acid [171], and endogenous molecules, such as microRNAs [70,151,166], UAP1L1 [158], and SIX1 [19], affect certain liver diseases through the O-GlcNAc metabolic pathway. Some of these are involved in the regulation of more than one signaling pathway. For example, NF-κB can be inhibited not only by curcumin to restrain hepatitis but also by OSMI-1 and corosolic acid to curb HCC. In addition, there are also molecular interactions; for example, YAP can upregulate O-GlcNAcylation to promote HCC [10], and corosolic acid inhibits HG/YAP/O-GlcNAcylation to inhibit HCC [171]. This suggests that the molecules involved in O-GlcNAc modification in the liver may constitute a complex network, which enables molecularly targeted therapy for one type of liver disease to simultaneously promote or block the development of other types of liver diseases. Likely, this may also be one of the reasons for drug resistance in various liver diseases. O-GlcNAcylation has both advantages and disadvantages in the regulation of liver pathological changes. On the one hand, O-GlcNAcylation can inhibit hepatic necrosis [8], suppress liver fibrosis [12,164], and resist infections [59,61,70,71]. On the other hand, it can promote liver inflammation [15,61,114], hepatic autophagy [26], hepatic steatosis [16,101], and HCC progression [20,129,164]. Liver fibrosis is one of the risk factors of HCC, but O-GlcNAcylation mainly inhibits the former and promotes the latter. Therefore, further research is needed to understand specific molecular mechanisms of O-GlcNAcylation in diverse settings. At the same time, more efforts are required to explore the detailed pathogenic mechanisms of related liver diseases. Among all kinds of liver diseases associated with O-GlcNAcylation, HCC might involve the largest proportion of molecules. As mentioned above, OGT plays a key role in the proliferation of HCC cells, and OGA affects the development and progression of HCC. This suggests that the OGT/OGA enzyme pair may be a potential target for HCC therapy. Since O-GlcNAcylation is more involved in regulating the expression of oncogenes, precisely targeting OGT/OGA and HBP flux may have greater anti-tumor efficacy. For this, some studies have been conducted, such as the combination of DOX and OSMI-1 [170].

The involvement of OGT in liver diseases is associated with many molecules and signaling pathways, but knowledge of its regulation in liver diseases is still limited. Therefore, a deeper understanding of the relevant molecular regulatory network in liver diseases will facilitate the development of more effective treatment of liver diseases in the future.

## Abbreviations

ACC, Acetyl-CoA carboxylase; ACSL4, Acyl-CoA ligase 4; AGER, Advanced glycosylation end product-specific receptor; AIMP2, Aminoacyl tRNA synthetase complex-interacting multifunctional protein 2; AKT, Protein kinase B; APAP, Acetaminophen; AS160, 160 kDa AKT substrate; ASCT2, Alanine/serine/cysteine transporter 2; Bcl-2, B-cell lymphoma 2; CA, Corosolic acid; CaMKII, Calcium/calmodulin-dependent kinase II; cAMP, Cyclic AMP; CAV1, Caveolin-1; CBS, Cystathionine-beta-synthase; CDK, Cyclin-dependent kinase; CEBPα, CCAAT/enhancer-binding protein alpha; CHOP, C/EBP homologous protein; ChREBP, Carbohydrate-responsive element-binding protein; Con A, Concanavalin A; dNTPase, Deoxy-ribonucleoside triphosphate; DOX, Doxorubicin; E. histolytica, Entamoeba histolytica; eIF2α, Eukaryotic translation initiation factor 2α; eIF4E, Eukaryotic initiation factor 4E; EMT, Epithelial-mesenchymal transition; ER, endoplasmic reticulum; EZH2, Enhancer of zeste homolog 2; FAS, Fatty acid synthase; FBPase, Fructose-1,6-bisphosphatase; FOXA2, Forkhead box protein A2; F6P, Fructose-6-phosphate; FXR, Farnesoid X receptor; GFAT, Glutamine Fru6p amidotransferase; GLUT1, Glucose transporter 1; GS, Glycogen synthase; GSH, Glutathione; GSK3β, Glycogen synthase kinase-3β; HBP, Hexosamine biosynthetic pathway; HBV, Hepatitis B virus; HCC, Hepatocellular carcinoma; HCV, Hepatitis C virus; HDAC1, Histone deacetylase-1; HG, High glucose; HIF-1α, Hypoxia-inducible factor 1α; HNF4α, Hepatocyte nuclear 4α; HSCs, Hepatic stellate cells; IRE1α, Inositol requiring enzyme 1α; KAT5, Lysine acetyltransferase 5; LD, Lipid droplet; L-PK, L-pyruvate kinase; LR, Liver regeneration; LT, Liver transplantation; LXRs, Liver X receptors; MCD, Methionine–choline-deficient; MLKL, Mixed lineage kinase domain-like; MMPs, Matrix metalloproteinases; NAFLD, Nonalcoholic fatty acid liver disease; NASH, Nonalcoholic steatohepatitis; OGA, O-GlcNAcase; O-GlcNAcylation, O-linked β-N-acetylglucosaminylation; OGT, O-GlcNAc transferase; PARP1, Poly (ADP-ribose) polymerase 1; PCK1, Phosphoenolpyruvate carboxykinase 1; PDGFRβ, Platelet-derived growth factor receptor β; PFKFB2, 6-phosphofructo-2-kinase/fructose-2,6-biphosphatase 2; PGC1α, Peroxisome proliferative-activated receptor gamma coactivator 1 alpha; PKA, Protein kinase A; PKCβII, Protein kinase C βII; PSAT1, Phosphoserine aminotransferase 1; RACK1, Receptor for activated C-kinase 1; RANBP2, RAN-binding protein 2; RIPK3, Receptor-interacting protein kinase 3; RUNX2, Runt-related transcription factor 2; SAMHD1, Sterile alpha motif and histidine/aspartic acid domain-containing protein 1; SCD1, Stearoyl-CoA desaturase1; Ser, Serine; SIX1, Sine oculis homeobox homolog 1; Sp1, Specificity protein 1; SREBP, Sterol regulatory element-binding protein; SRF, Serum response factor; TFF2, Trefoil factor 2; Thr, Threonine; UAP1, UDP-N-acetylglucosamine pyrophosphorylase 1; UAP1L1, UAP1-like-1; UDP, Uridine 5′-diphosphate; URI, Unconventional prefoldin RPB5 interactor; USP2A, Ubiquitin-specific protease-2a; XBP1, X-box-binding protein 1; YAP, Yes-associated protein; YB-1, Y-box binding protein 1; α-SMA, α smooth muscle actin.
